# The mutual patterning between the developing nephron and its covering tissues—valid reasons to rethink the search for traces left by impaired nephrogenesis

**DOI:** 10.1186/s40348-021-00120-0

**Published:** 2021-08-17

**Authors:** Will W. Minuth

**Affiliations:** Institute of Anatomy, University of Regensburg, D - 93053 Regensburg, Germany

**Keywords:** Fetal human kidney, Nephrogenic zone, Impairment of nephrogenesis, Nephron development, Covering tissues

## Abstract

**Background:**

The impairment of nephrogenesis can cause the termination of nephron formation in preterm and low birth weight babies. This leads to oligonephropathy with severe health consequences in later life. Although many clinical parameters are known, surprisingly little information is available regarding the initial damage on the developing nephron. Equally astounding, the first morphological data regarding the specifics of nephron formation in the nephrogenic zone of the fetal human kidney during late gestation has only been published within the past few years. In this context, it was observed that each stage of nephron anlage is surrounded by a specific set of tissues. Although highly relevant for the normal progress of nephron formation, the mutual patterning has not been systematically described.

**Results:**

To contribute, the different stages of nephron anlage in the nephrogenic zone of the fetal human kidney during late gestation were screened by the optical microscope and documented by images. Following this, magnifications (28 × 18 cm) were produced to trace the contours of the developing nephron and its covering tissues. The resulting sketches, almost true to scale, were scanned, edited, and processed by a design program. As a base, first the individual position, size, and shape of the nephrogenic niche, pretubular aggregate, renal vesicles, comma- and S-shaped bodies are presented. Secondly, their structural relations to the renal capsule, collecting duct ampulla, perforating radiate artery, and expanding interstitium are shown. Third of all, the focus is on less considered configurations, such as site-specific approximation, local distancing, punctual adhesion, integration, separation, delamination, formation of congruent and divergent surfaces, and folding and opening of interstitial clefts.

**Conclusions:**

The present contribution illuminates the mutual patterning between the developing nephron and its covering tissues. It is indispensable to know about the microanatomical relations, in order to identify whether the noxae impairing nephrogenesis targets only the developing nephron or also its covering tissues as interacting and controlling instances.

## Background

### Impairment of nephrogenesis

A series of noxae different in molecular compositions can provoke an early termination of nephron formation in preterm and low birth weight babies [1, 2]. While the clinical parameters have been intensely analyzed, comparatively little information is available with regards to the affected stages of nephron anlage, the concrete cell targets of noxae, and the molecular processes leading to pathological alterations in the outer cortex of the fetal human kidney. The situation is further complicated by the fact that the microscopic assessment of specimens needs special attention, since the target region of noxae is complexly built up. Covered by the renal capsule, it consists of fetal, maturing, and matured tissue layers [3]. This reflects a structural gradient, in which the process of nephron formation is integrated. It starts near the inner side of the renal capsule, lines perpendicular to it through the external nephrogenic zone, the subjacent maturation zone, and then the matured zone.

### Detection of long-term effects

The transiently appearing stages of nephron anlage, which are restricted to the nephrogenic zone, are relevant for the impairment of nephrogenesis. Beginning with the nephrogenic niche, these include the successively developing pretubular aggregate, renal vesicles, and comma- and S-shaped bodies. Using the rodent kidney as an experimental model, it was demonstrated that the exposure to noxae targets the nephrogenic niche resulting in a low expression of morphogenic molecules such as Gfrα1, Gdnf, Bmp4, Pax2, and Six2 [4]. Surprisingly, comparable data dealing with a damage on the early stages of nephron anlage in the fetal human kidney is lacking. Instead, the limited data available shows that the width of the nephrogenic zone in gestational controls is 150 µm, while in preterm babies, it is significantly smaller at 100 µm [5]. Furthermore, the lack of basophilic S-shaped bodies in the nephrogenic zone of preterm babies was reported [6]. However, in these publications, information dealing with the pathological alterations of the tissues covering the starting nephron is not provided.

Frequently mentioned is the detection of atypical glomeruli including a dilated Bowman’s space and a shrunken glomerular taft [5] and/or a decrease in the number of glomeruli [7]. However, these pathological findings do not concern primarily the stages of nephron anlage in the nephrogenic zone but the maturing nephron in the underlying maturation zone. Surprisingly, no previous related publications provide a clear proof on whether the noxae impairing nephrogenesis has a direct impact on the developing nephron, or whether it is indirect and caused by a disturbance of the covering tissues and related molecular interactions.

### Rather unexplored terrain

Although surprisingly, the analysis of pertinent literature further revealed that the role of the nephrogenic zone in the fetal human kidney in nephron formation and consequently as the primary target region for noxae impairing nephrogenesis has been hardly explored. Before this background, it is understandable that the contained stages of nephron anlage and the shaping of the nephron were only introduced over the past few years as the first systematic morphological information dealing with the specifics of the nephrogenic zone [8–10]. The results generated indicate that each stage of nephron anlage is the subject of an individual positioning, orientation, and shaping. A further important finding in this context is that during the progress of development, not only the individual stage of nephron anlage but also the corresponding covering tissues are specifically changing. As a consequence, the aim of the present morphological investigation is to demonstrate the precise site of nephron formation, to inform about important coordinates, and to document the mutual patterning of its covering tissues.

### Zones in the outer cortex

Noxae impairing nephrogenesis targets the outer cortex of the fetal human kidney during late gestation. Covered by the renal capsule, it is built up by transverse layers of embryonic, maturing, and matured parenchyma and stroma. In the external nephrogenic zone, the process of nephron formation starts [9] and can be recognized by the transient stages of nephron anlage [10, 11]. In the subjacent maturation zone, the conversion of the S-shaped body as the last stage of nephron anlage into the lasting nephron occurs. This process comprises the elongation, spatial extension, and functional differentiation of the individual nephron segments. In the underlying matured zone, the typical morphological features of the definitive nephron are established.

### Location of initial nephron morphogenesis

Most relevant in the search for initial imprints left by the impairment of nephrogenesis is the external nephrogenic zone. This can be seen underneath the renal capsule as a thin strip of embryonic parenchyma and stroma [9, 12]. In the fetal human kidney, the outer border is in contact with the inner side of the renal capsule. The inner border has been previously defined as a “dotted” line, which meets the proximal (medulla-orientated) pole of the side by side aligned S-shaped bodies in a transverse orientation [5, 13]. Furthermore, it has been defined that the vertical distance between the renal capsule and the proximal pole of an S-shaped body is 150 µm. At its inner border, the nephrogenic zone is facing the subjacent maturation zone. Components of the nephrogenic zone are the nephrogenic and stromal progenitor cells, the transiently appearing stages of nephron anlage such as the nephrogenic niche, pretubular aggregate, renal vesicles, and comma- and S-shaped bodies. In addition, the ureteric bud-derived collecting duct (CD) ampullae are extending at the distal endings of the collecting duct tubules. The perforating radiate arteries line in vertical direction. In the arising interstitium and along the microvascular system, numerous macrophages are visible [14].

### Individual site of nephron formation

**Fig. 1:**
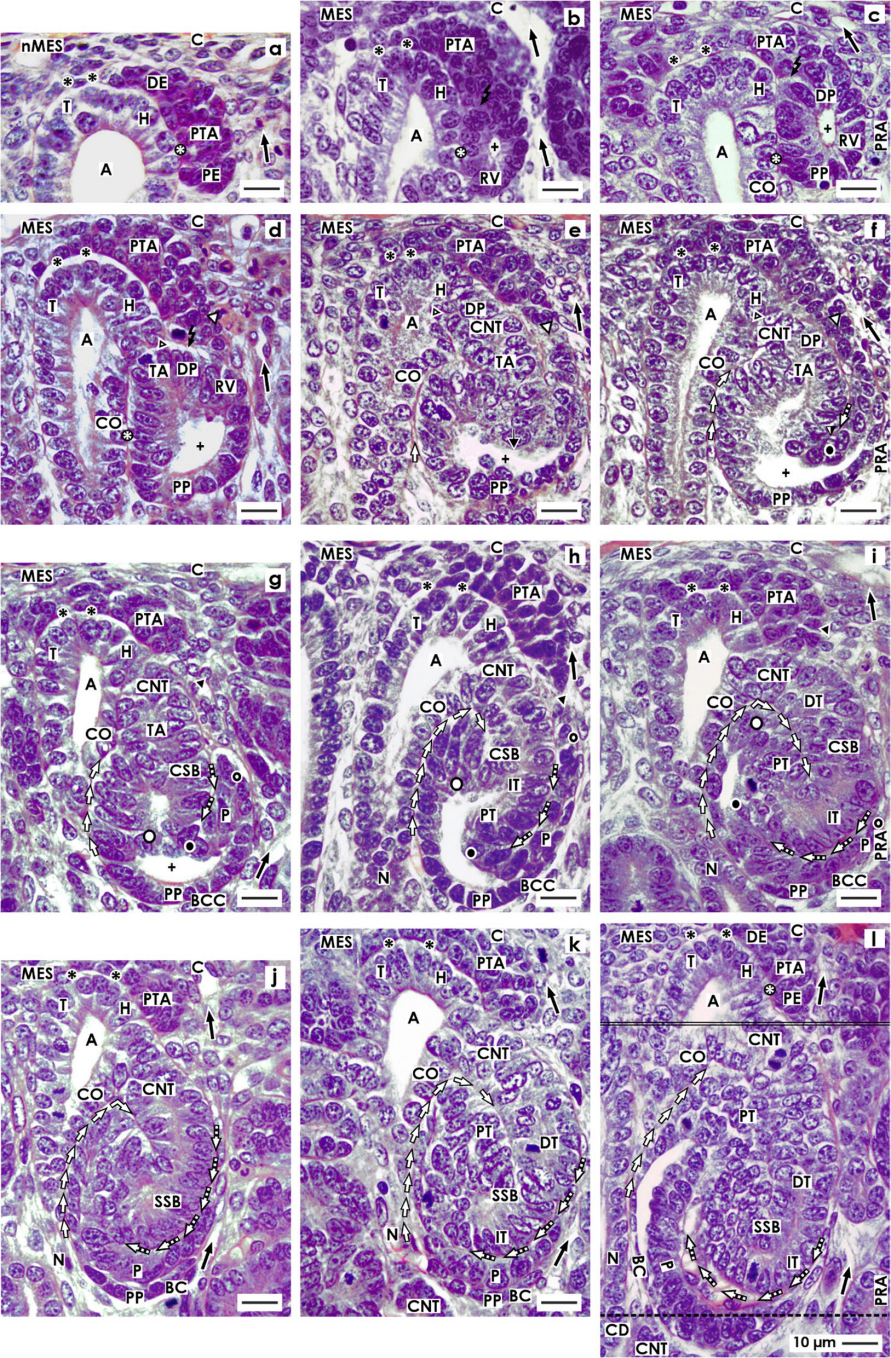
View of the stages of nephron anlage, restricted to the nephrogenic zone in the fetal human kidney during late gestation through the optical microscope. The development of a single nephron takes place in a nephrogenic compartment. Within its district of progenitor cell recruitment, **a** the nephrogenic niche (black asterisk) and the pretubular aggregate (PTA), **b** the mesenchymal to epithelial transition, and **c** the appearance of the primitive renal vesicle (RV) is registered. In its underlying area of nephron shaping the **d** mature, **e** extending, and **f** extended renal vesicles are seen. Then, the **g** early, **h** mid, and **i** late comma-shaped bodies (CSB) and the **j** early, **k** mid and **l** late S-shaped bodies (SSB) develop. Renal capsule (C), collecting duct (CD) tubule, CD ampulla (A) composed of a tip (T), head (H), conus (CO) and neck (N), perforating radiate artery (PRA, short black arrow), lumen of the arising nephron + . Transverse double line: border between the district of progenitor cell recruitment and the area of nephron shaping. Dotted line: border between the external nephrogenic zone and the subjacent maturation zone. Individual images have been previously published [10]

When vertical lines are drawn, the nephrogenic zone can be divided into numerous nephrogenic compartments [10, 11, 15]. These are aligned side by side in a row. Within a nephrogenic compartment, the development of a single nephron from the recruitment of progenitor cells to the late S-shaped body takes place (Fig. 1). At the top, each of the nephrogenic compartments is covered by the renal capsule. Its medial border can be recognized by a vertically lining CD ampulla. The lateral border is defined by a perforating radiate artery, which lines vertically towards the renal capsule. The border at the base is the proximal pole of the respective stage of nephron anlage up to the S-shaped body. This is positioned above the connecting tubule of an earlier developed nephron.

**Fig. 2:**
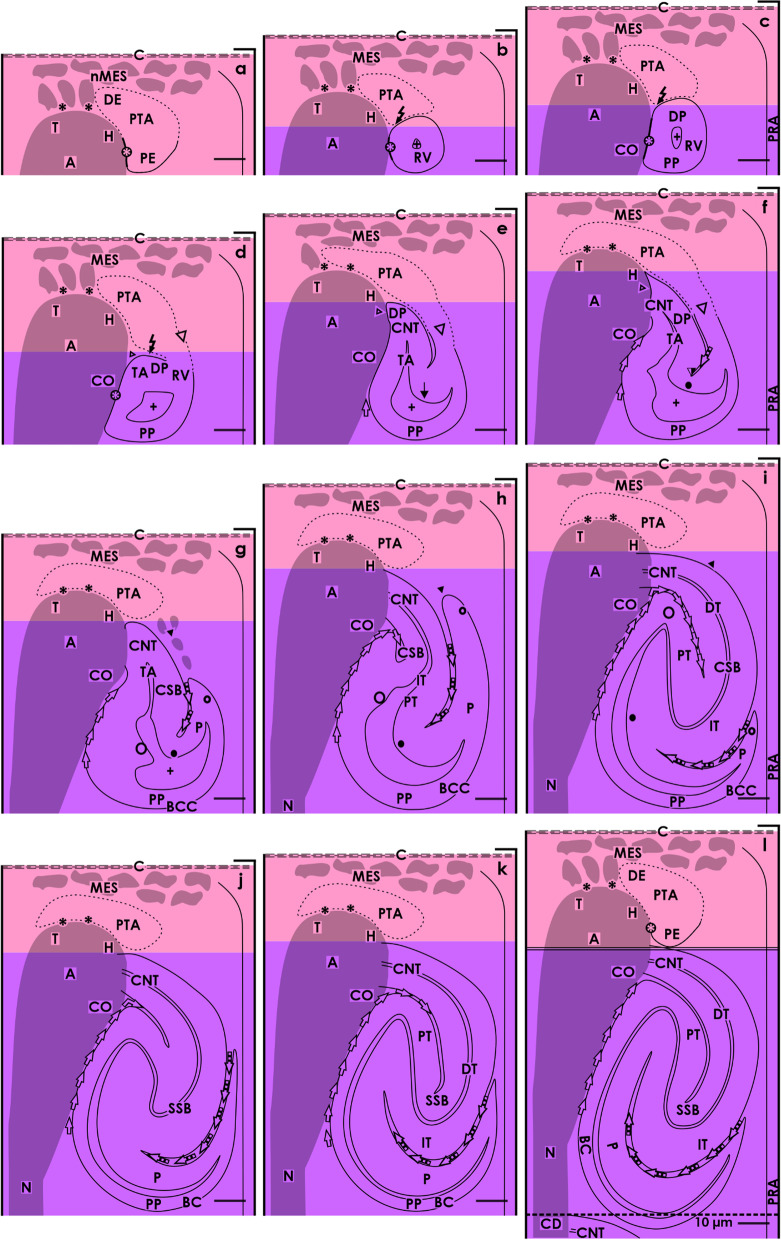
Graphical sketches depicting the translocation of the developing nephron in a nephrogenic compartment of the fetal human kidney during late gestation. In the district of progenitor cell recruitment (light) the **a** nephrogenic niche (black asterisk) with the pretubular aggregate (PTA), **b** the mesenchymal to epithelial transition, and **c** the primitive renal vesicle (RV) are visible. In the underlying area of nephron shaping (dark), the **d** mature, **e** extending, **f** extended renal vesicles, the **g** early, **h** mid, and **i** late comma-shaped body (CSB) and the **j** early, **k** mid, and **l** late S-shaped body (SSB) are registered. While the district of progenitor cell recruitment maintains a constant size, the area of nephron shaping continues to expand during the formation of **c** the matured renal vesicle (15 × 20 µm) to **l** the late S-shaped body (100 × 100 µm). Renal capsule (C), collecting duct (CD) tubule, CD ampulla (A), perforating radiate artery (PRA, short black arrow), and lumen of the arising nephron +

Within a nephrogenic compartment, a certain number of progenitor cells of both the presently developing nephron and the future generation are produced. This not only requires the number of progenitor cells to be controlled, but also to ensure that they remain in the correct place and that a percentage is transferred into the state of competence for induction [16]. After the exchange of morphogenic molecules in the nephrogenic niche, the actual morphogenesis can begin. This stage can be recognized by a cell aggregation and the subsequent formation of the pretubular aggregate (Fig. 1a), the mesenchymal to epithelial transition (Fig. 1b), and the development of the primitive renal vesicle (Fig. 1b) next to the tip and then the head of the CD ampulla. When the mature renal vesicle separates from the pretubular aggregate (Fig. 1c), in order to begin with the shaping of the nephron (Fig. 1d–l), the backdrop of development is shifting to the conus of the CD ampulla. For this reason, it is important to differentiate between the above-positioned district of progenitor cell recruitment and the underlying area of nephron shaping (Fig. 2).

### District of progenitor cell recruitment

The district of progenitor cell recruitment represents a prone rectangle, which principally maintains its size during the formation of a nephron (Fig. 2a) [10, 11]. It is in direct contact with the inner side of the renal capsule and shows a transverse length of about 80 µm. The medial and the lateral borders are 50 µm in length on average. Its lower transverse border lines through the section border between the head and conus of the related CD ampulla.

The tissue configuration in the district of progenitor cell recruitment is remarkable. When histological sections of the pretubular aggregate are observed, the available space between the tip of a CD ampulla and the inner side of the renal capsule is surprisingly narrow (Figs. 1a and 2a). Only 2 to 3 layers of mesenchymal progenitor cells are seen here [8]. For the rodent kidney, it was shown that these layers are arranged in a topological order. The nephrogenic progenitors face the tip of a CD ampulla, while the stromal progenitors are distributed near the inner side of the renal capsule [17].

At the tip of a CD ampulla, the nephrogenic niche is formed as the first stage of nephron anlage. In the immediate vicinity, as well the aggregation of induced nephrogenic mesenchymal cells, the formation of the pretubular aggregate (Figs. 1a and 2a), the mesenchymal to epithelial transition (Figs. 1b and 2b), and the appearance of the primitive renal vesicle (Figs. 1c and 2c) is noticed. The section border between the head and the conus of the CD ampulla determines the further process. Here, a link with the primitive renal vesicle takes place to fix the future connecting tubule (CNT). During this process, at the medial part of its distal pole, the renal vesicle is separated from the pretubular aggregate, while the lateral part remains connected via a two-layered progenitor cell strand (Figs. 1c, d and 2c, d).

### Area of nephron shaping

This area represents an extending quadrate, in which the development from the mature renal vesicle (Figs. 1c and 2c) to the late S-shaped body (Figs. 1l and 2l) is successively progressing. At the beginning, the area of nephron shaping resembles the small size of a mature renal vesicle (ca 20 × 20 µm), but finally reaches vertical and transverse lengths of up to 100 µm each during the development of the S-shaped body [10, 11].

The upper border of the area of nephron shaping lines transversely at the section border between the head and conus of the related CD ampulla. It also meets the attachment site of the mature renal vesicle on the CD ampulla, as the proximal end of the pretubular aggregate (Figs. 1d and 2d). More laterally, the upper border crosses the mesenchymal progenitor cell strand, which connects the proximal end of the pretubular aggregate with the extending (Figs. 1e and 2e) and extended (Fig. 2e, f) renal vesicles. In the early comma-shaped body, it crosses the site where the separation from the pretubular aggregate occurs (Figs. 1g and 2g). The medial border lines along the conus of the CD ampulla, while the lateral border is near a vertically lining perforating radiate artery. The lower transverse border lies between the external nephrogenic zone and the subjacent maturation zone. As a consequence, it crosses the proximal pole as well as that of the developing renal vesicles (Figs. 1d–f and 2d–f) and the comma- (Figs. 1g–i and 2g–i) and the S-shaped bodies (Figs. 1j–l and 2j–l). As previously mentioned, the respective proximal pole rests near the transversely lining connecting tubule of an earlier developed nephron. The configuration resembles an immovable base, which blocks the spatial expansion of the developing nephron near the proximal pole, but enables the lifting of the entire nephrogenic compartment step by step together with the renal capsule. Finally, regarding microscopic specimens within the area of nephron shaping, beside the attachment site of the future connecting tubule (CNT) at the section border between the head and conus of the CD ampulla either mature, extending, or extended renal vesicles (Figs. 1d–f and 2d–f), a comma-shaped body (Figs. 1g–i and 2g–i) or a S-shaped body (Figs. 1j–l and 2j–l) can be observed.

### Contours at the transient stages of nephron anlage

**Fig. 3:**
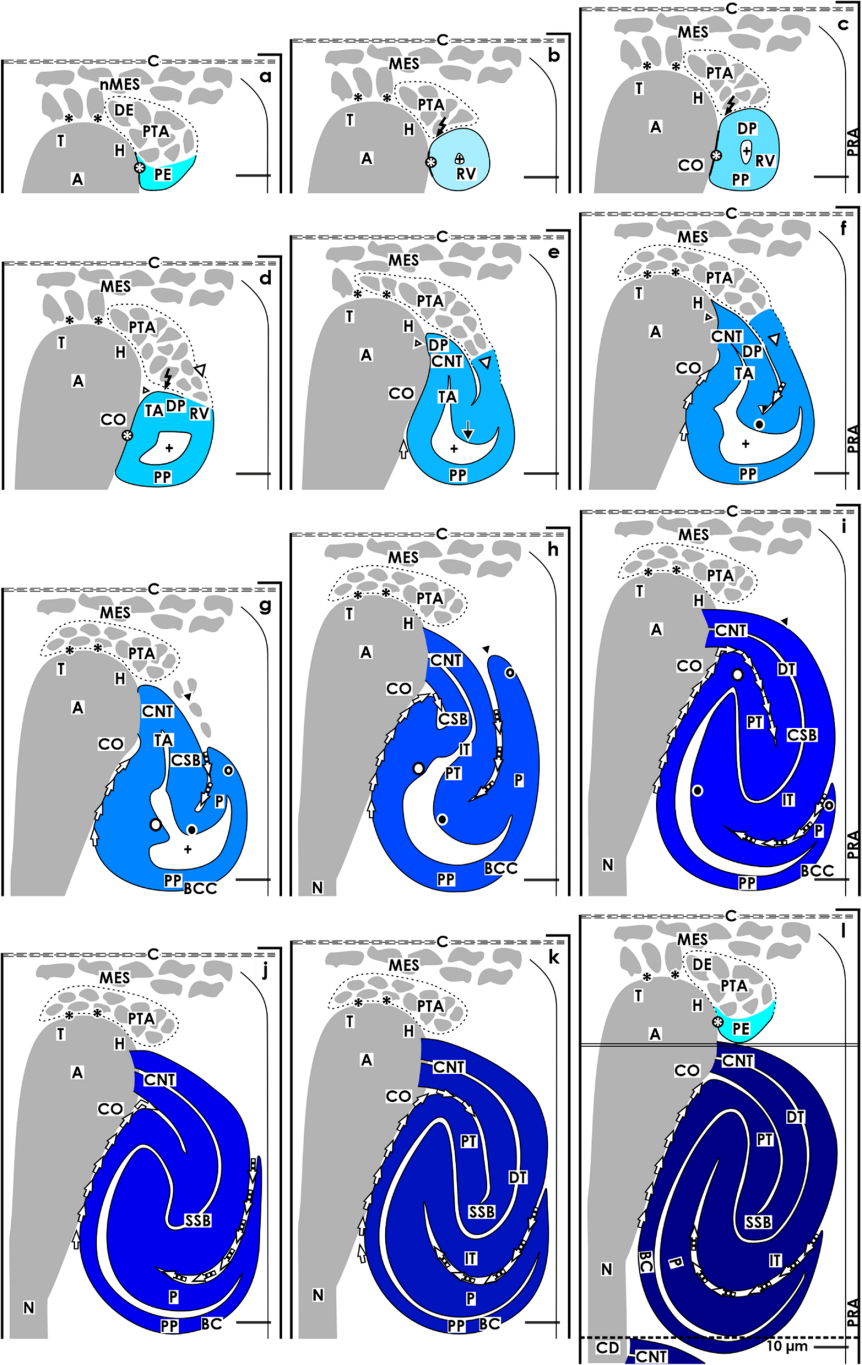
Graphical sketches illustrate key developmental points of a nephron in the fetal human kidney during late gestation. **a** Besides the mesenchymal progenitor cells, also the medial aspect of the pretubular aggregate (PTA) is separated from the tip and head of a CD ampulla by a clear interface (black asterisk). **b** During the mesenchymal to epithelial transition and **c** the subsequent appearance of the primitive renal vesicle (RV) a close adhesion (white asterisk) is noticed at the section border between the head and conus of the CD ampulla. Following this, the medial part of the distal pole (DP) at a primitive renal vesicle (RV) starts to separate from the pretubular aggregate. **d** The lateral part of the distal pole at the mature renal vesicle stays connected with the pretubular aggregate via a progenitor cell strand (white arrow head). In the **e** extending and **f** extended renal vesicles the elongation of the tubule anlage (TA) provokes the formation of an inner fold (big black/white point). An interstitial cleft (white/black arrows) elongates between the CD ampulla and the medial aspect of the renal vesicles. Interestingly, between the future connecting tubule (CNT) and the progenitor cell strand connecting the renal vesicle with the pretubular aggregate also an interstitial cleft extends. **g** During formation of the early comma-shaped body, the progenitor cell strand connected with the PTA dissolves, enabling the lateral fold (small black/white point) to become visible. **h** In the mid comma-shaped body, a vertical interstitial cleft expands between the tubule anlage and the future glomerulus. **i** In the late comma-shaped body, this changes direction from vertical to transverse. In the **j** early, **k** mid, and **l** late S-shaped bodies the glomerulus anlage remains positioned at the proximal pole (PP), while the distal pole is near the CNT, forming a contact with the border between the head and conus of the CD ampulla. A vertical interstitial cleft elongates between the conus of the CD ampulla and the medial aspect of the S-shaped body. At the lateral aspect, the opening of the transverse interstitial cleft between the tubule and glomerulus anlage is positioned near a perforating radiate artery (PRA, short black arrow). Renal capsule (C), collecting duct (CD) tubule, CD ampulla (A), and lumen of the arising nephron +

For the fetal human kidney during late gestation, it was demonstrated that the initial formation of a nephron takes place in a nephrogenic compartment located in the nephrogenic zone [10]. In microscopic specimens, this is recognized by the transient stages of nephron anlage including the nephrogenic niche, pretubular aggregate, renal vesicles, and comma- and S-shaped bodies, which are present at this point (Figs. 1 and 3).

### Nephrogenic niche, pretubular aggregate, and primitive renal vesicle

The formation of a nephron is initiated by the process of induction. This takes place in a nephrogenic niche and within close proximity to the inner side of the renal capsule [8]. During this process, the most inner layer of the nephrogenic progenitor cells faces the basal aspect of the epithelial progenitor cells contained in the ureteric bud-derived tip of a CD ampulla. A series of morphogenic molecules such as Gdnf, Wnts, Fgfs, and Bmps are then exchanged [16]. It can be recognized that in this situation, the nephrogenic mesenchymal progenitor cells are separated from the tip of the CD ampulla by a clear interface (Figs. 1a and 3a) [10].

When the process of induction is successful, the nephrogenic mesenchymal progenitor cells increase in size, their shape becomes angular, and the intercellular spaces enlarge to aggregate firstly along the tip and then head of the CD ampulla. This leads to the formation of the pretubular aggregate, which looks like a teardrop (Figs. 1a and 3a). Its thin distal end is orientated towards the renal capsule so that it remains in contact with the inner layer of the nephrogenic mesenchymal progenitor cells. In contrast, the thickened proximal end points towards the medulla. A clear interface is visible between the distal end of the pretubular aggregate and the tip of the CD ampulla. However, between the proximal end of the pretubular aggregate and the head of the CD ampulla, a close adhesion is noticed. Uneven interstices between the cells and a smoothening of the surface at the proximal end of the pretubular aggregate are first signs for the mesenchymal to epithelial transition (MET) (Figs. 1b and 3b).

As a result, the primitive renal vesicle, with a polarized epithelium and a small lumen, becomes visible (Figs. 1c and 3c). In parallel, a transverse but incomplete separation of the renal vesicle from the pretubular aggregate is seen at the border between the head and conus of the CD ampulla. The process of separation extends transversely as far as the middle of the pretubular aggregate, so that the lateral part of the renal vesicle remains connected at this timepoint with the pretubular aggregate via a two-layered progenitor cell strand.

### Mature, extending, and extended renal vesicles

After the development of the primitive renal vesicle, the setting is translocated from the district of progenitor cell recruitment to the underlying area of nephron shaping (Figs. 1d–f and 3d–f). In the rodent kidney, a distinction is made between the primitive, mature, and extending renal vesicles 1 to 5 [17–19]. Comparable experimental information in the fetal human kidney is not currently available. However, on the microscopic specimens, a site of considerable experimental interest is the distal pole of the mature renal vesicle (Figs. 1d and 3d). Its medial part detaches from the pretubular aggregate to be fixed on the CD ampulla at the section border between its head and conus. At this site, the future connecting tubule (CNT) and the tubule anlage become visible. Noticeable, the inner part of the distal pole remains detached from the overlying pretubular aggregate, while the lateral part remains connected with the pretubular aggregate via the two-layered progenitor cell strand. Due to the epithelial folding, the lumen in a mature renal vesicle is uneven, and it is V-shaped at the distal pole and rounded off at the proximal pole. Between the conus of the CD ampulla and the medial aspect of the renal vesicle, a vertically lining interstitial cleft starts to develop.

In the extending renal vesicle, the lumen is changing (Figs. 1e and 3e). While remaining rounded at the proximal pole, the vertical elongation of the tubule anlage causes a protrusion at the distal pole. Following this, the renal vesicle continues to extend not only lengthwise but also widthwise. The interstitial cleft then begins to elongate vertically between the conus of the CD ampulla and the medial aspect of the renal vesicle.

In the extended renal vesicle, the future CNT invades the epithelium of the CD ampulla in order to connect with it at the section border between its head and conus (Figs. 1f and 3f). The interstital cleft between the conus of the CD ampulla and the medial aspect of the renal vesicle elongates vertically up to the CNT. The lateral part of the distal pole is still connected with the pretubular aggregate via a two-layered progenitor cell strand. The tubule anlage elongates in a vertical direction to form an inner fold. The medial leg lines up with the epithelial cell strand of the presumptive CNT to end at the head of the CD ampulla. The lateral leg of the fold is part of the progenitor cell strand, which is connected with the pretubular aggregate. An oblique interstitial cleft in form of a narrow pocket becomes visible.

### Comma-shaped body

During the development of the early comma-shaped body, the two-layered mesenchymal progenitor cell strand between its distal pole and the overlying pretubular aggregate is dissolving (Figs. 1g and 3g) [10]. Henceforth, the developing nephron can no longer partake in the progressive recruitment of progenitor cells [20]. The tubule anlage elongates vertically towards the center of the comma-shaped body. This causes the medial, inner as lateral folds to elongate. As a result, the comma-shaped body expands in vertical direction. At its proximal pole, the glomerulus including the Bowman’s capsule develops histo-typical features.

During the formation of the mid comma-shaped body, the epithelium of the CNT fuses with the epithelium of the CD ampulla at the border between its head and conus (Figs. 1h and 3h). The interstitial cleft between the conus of the CD ampulla and the medial aspect of the comma-shaped body continues to extend in length. In addition, an elongation of the proximal tubule segment is observed. The visceral epithelial cell layer transforms into the podocytes.

In the late comma-shaped body, the tubule segments further extend by meandering (Figs. 1i and 3i). At the medial aspect, the cleft between the conus of the CD ampulla and the comma-shaped body changes direction by expanding along the CNT. At the lateral aspect, the cleft between the proximal tubule segment and the visceral epithelial cell layer including the podocytes is changing direction from vertical to transverse. Consequently, it opens laterally and in the close proximity of a vertically lining perforating radiate artery. This constellation enables an afferent arteriole to invade the developing glomerular tuft via a shortcut.

### S-shaped body

The S-shaped body is the last stage of nephron anlage, which develops in the nephrogenic zone and has been analyzed in both the rodent and human kidney [21, 22]. The early S-shaped body still develops perpendicular to the renal capsule (Figs. 1j and 3j). At its distal pole, the CNT completes the connection with the CD ampulla at the section border between its head and conus, while at its proximal pole, the glomerulus and the Bowman’s capsule present typical morphological features. Both structures are separated from the tubular portion by a transversely lining interstitial cleft. Inside this cleft, the glomerular tuft, the intra- and extraglomerular mesangium as the capillary network are establishing themselves. It opens at the deep lateral aspect of the S-shaped body near a vertically lining perforate radiate artery.

At the medial aspect, the mid S-shaped body is separated from the conus and neck of the CD ampulla by a vertically lining interstitial cleft (Figs. 1k and 3k). It appears that the Bowman’s capsule and the conus of the CD ampulla form nearly congruent surfaces. At its proximal pole, the glomerulus extends causing the overlying interstitial cleft to elongate and change direction from vertical to transverse.

The late S-shaped body expands mainly in a vertical direction (Figs. 1l and 3l). At its proximal pole typical morphological features of the glomerulus are visible. The interstitial cleft between the developing glomerulus and the overlying tubular portion further extends so that a network of capillaries and the intraglomerular mesangium can establish. After reaching the interior of the S-shaped body it changes direction from transverse to sickle-shaped. In contrast, the interstitial cleft between the conus, and the neck of the CD ampulla and the medial aspect of the S-shaped body respectively, lines vertically to the CNT and then horizontally so that it is reaching finally the geometric center of the S-shaped body.

## Methods

In order to analyze morphological structures or to search molecular traces left by the impairment of nephrogenesis, one must focus on the different stages of nephron anlage, such as the nephrogenic niche, pretubular aggregate, renal vesicles, and comma- and S-shaped bodies. In a fetal human kidney during late gestation, these are restricted to the nephrogenic zone, which extends as a thin strip of parenchyma and stroma along the inner side of the renal capsule. It is obvious that during the histological preparation, it is vital to prevent a damage to this region. For this reason, a fetal human kidney is held only on the hilum, and the touching of the renal capsule with fine forceps is avoided [9]. Furthermore, for obtaining comparable perspectives in the histological sections, a fixed kidney is cut from the renal capsule towards the papilla of a lobe. Following this strategy, the section plane lines along the axis of the vertically running collecting duct (CD) tubules and perpendicular to the renal capsule. When this advice is followed, the renal capsule and the underlying nephrogenic zone with the contained stages of nephron anlage become visible in a comparable perspective.

The illustrations of fetal human kidneys, as shown here, were obtained from specimens of gestational age between weeks 16 and 18 as well as other later gestational times. They were selected from the stock of preparations used for the Course of Microscopic Anatomy for Medical Students at the University of Regensburg/Germany. According to routine methods, the tissue blocks were fixed in paraformaldehyde solution and embedded in paraffin wax. Following this, sections of 5 µm thickness were cut and stained with hematoxylin–eosin solution for analysis by the optical microscope. Screening of the stained sections was performed by a Leica DM750 microscope (Leica Microsystems, Wetzlar, Germany). The stages of nephron anlage were analyzed with a HI Plan 63x/0.75 objective lens. Images were taken with a Basler Microscopy Pulse 5.0 camera (Basler AG, Ahrensburg, Germany).

More than 3000 images were available, which had previously been analyzed for an investigation studying the shaping of the nephron [10]. Multiple representative images, which illustrate basic stations during initial nephron development, were selected from this collection. The aim of the present investigation was to focus not only on the developing nephron, but to also show the covering tissues and the mutual patterns between both. For this purpose, as well photographic illustrations as true to scale graphical sketches were produced.

To meet the defined criteria and to be as precise as possible, a morphometric computer program was used. However, due to inaccuracy, nebulization, and misinterpretation, the analysis failed. As a necessary consequence, a series of sketches had to be made. First, the eligible microscopic images were magnified (28 × 18 cm). Then, the contour lines of the different stages of nephron anlage and of the covering tissues were marked 1:1 by hand with a pencil. Next, the generated contours were scanned, edited, and processed by the design program CorelDRAW X7 (Corel Corporation, Munich, Germany). The same program was used to insert the necessary labels and to identify metric parameters in the microscopic images or generated sketches.

Finally, to visualize the spatial and temporal changes, mounted image plates were produced. As it is indicated in the illustrations, the proximal pole (fixed point) of the currently developing nephron rests, as in the kidney, near the connecting tubule of an earlier developed nephron, while the distal pole (mobile point) of the currently developing nephron shifts radially, step by step, together with the overlying renal capsule.

To indicate specific sites in the images and graphical sketches shown here, a consistent nomenclature including numerous specific terms, abbreviations, and inserts was used consistent with earlier publishing [10, 11]. These are allocated to the explained structures as shown in Table 1.

**Table 1:**
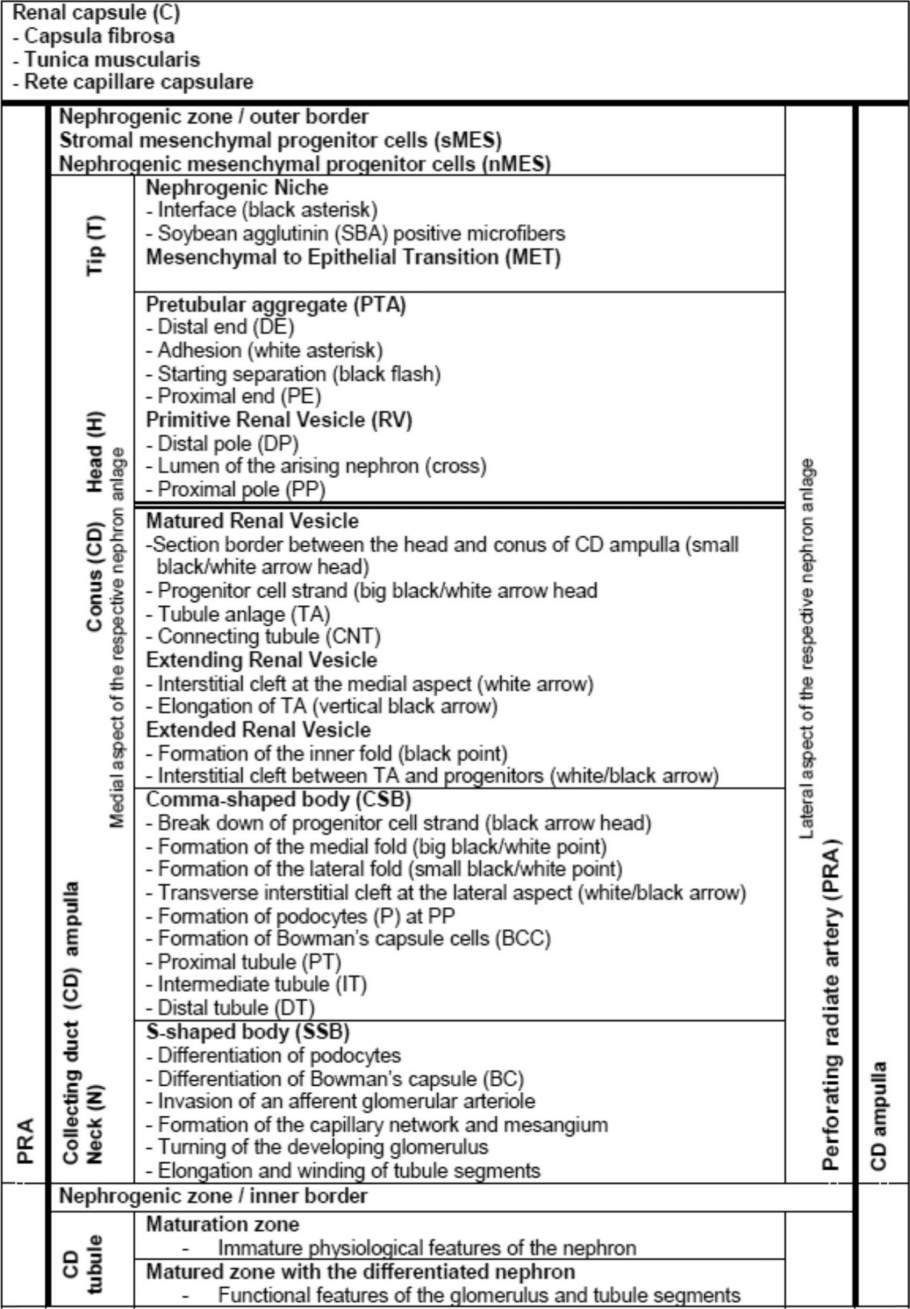
Terms and abbreviations are allocated to the structural conditions of the outer cortex in the fetal human kidney during late gestation as it was previously published [10, 11]. The thick black lines depict the border of a nephrogenic compartment. The separation between the district of progenitor cell recruitment and the area of nephron shaping is indicated by a transverse double line. The dotted line marks the border between the external nephrogenic zone and the subjacent maturation and matured zones

## Results

Frequently, the formation of a nephron is portrayed as the one and only existing objective in the developing kidney. However, a look onto microscopic specimens of the nephrogenic zone in late gestation of the fetal human kidney reveals that it is enclosed by other parenchymal and stromal components (Fig. 1). The remaining unanswered questions consist of their appearance and arrangement as well as whether they maintain a rigid frame or show a mutual patterning from the nephrogenic niche to the S-shaped body.

### The renal capsule near the distal pole

**Fig. 4:**
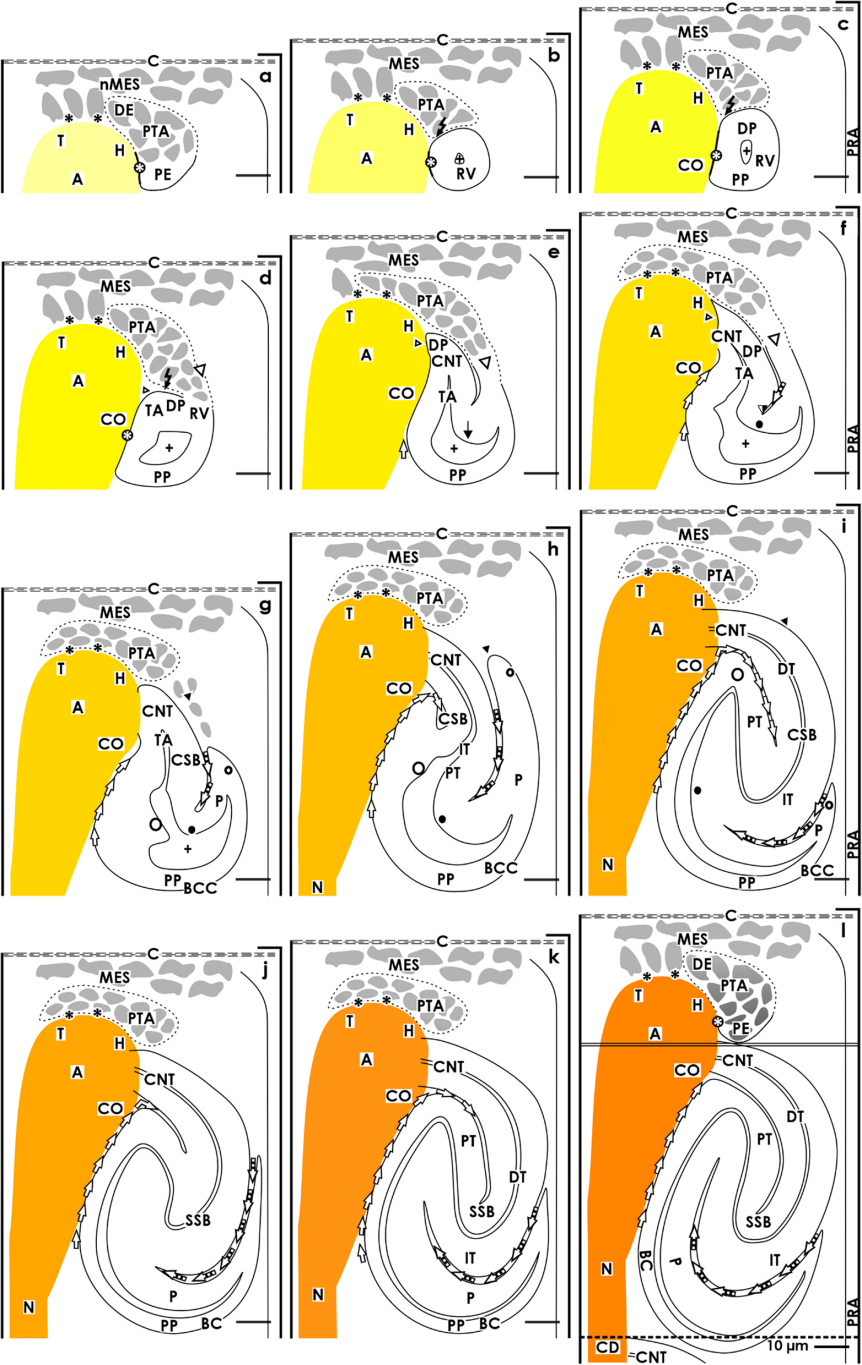
Graphical sketches show the structural relations between the CD ampulla and the developing nephron in the fetal human kidney during late gestation. The distal end of a collecting duct (CD) dilates to form a CD ampulla (A), which expands radially. It consists of a tip (T) pointing to the renal capsule (C), head (H), conus (CO), and neck (N). There are only 2 to 3 layers of mesenchymal progenitor cells (MES) separating the renal capsule from the tip. **a** A striking interface (black asterisk) separates the epithelial progenitor cells in the tip of a CD ampulla from the innermost layer of nephrogenic mesenchymal progenitor cells. This special arrangement indicates the center of a nephrogenic niche. The forming pretubular aggregate (PTA) is first found close to the tip and then head of the CD ampulla. **b** The mesenchymal to epithelial transition and the initial separation of the primitive renal vesicle occurs at the border between the head and conus of the CD ampulla. **c** At this point, its medial aspect adheres so that the future connecting duct tubule (CNT) can form. Although separated by an interstitial cleft, the medial aspects of the **d** mature, **e** extending, **f** extended renal vesicles, the **g** early, **h** mid, and **i** late comma-shaped bodies (CSB) as well as the **j** early, **k** mid, and **l** late S-shaped bodies (SSB) are near the concave conus of the CD ampulla. The proximal pole (PP) of the S-shaped body is near its neck and faces the underlying maturation zone. In contrast, the lateral aspects of the renal vesicles, the comma- and the S-shaped bodies are covered by the loose mesenchyme and face a vertically lining perforating radiate artery (PRA, short black arrow). Collecting duct (CD) tubule, CD ampulla (A) composed of a tip (T), head (H), conus (CO) and neck (N), and lumen of the arising nephron +

In the fetal human kidney during late gestation, the initial formation of a nephron starts near the renal capsule and is restricted to the nephrogenic zone (Figs. 1 and 4) [23]. Often it is regarded as a more or less primitive envelope, which protects the developing kidney from undesirable mechanical and physiological influences. However, it is built up according to the developmental needs and contains a series of progenitor cells, and most interestingly, it controls the early patterning of the nephron [15, 23]. In microscopic specimens, it can be seen that the renal capsule consists of a capsula fibrosa including several strata. Contained are type I, type III collagens, and aminopropeptide type III procollagen, which establishes a link with the extracellular matrix contained in the outer cortex [24]. This kind of molecular construction acts like a padding enabling a certain elasticity between the neighboring structures. Surprisingly, little attention has been given to the tunica muscularis. As the innermost (visceral) layer of the renal capsule, it is in direct contact with the outer side of the nephrogenic zone and the here present stromal and nephrogenic progenitor cells [17]. At the transition into the nephrogenic zone, atypical smooth muscle cells develop, which show numerous projections. Some of these are covered by a basal lamina, while on others, only a faint glycocalix is recognized [25]. Most remarkably, the projections establish multiple contacts with neighboring cells, so that an extracellular tunnel network is formed. Although it has not been experimentally investigated, it could be speculated that this serves to direct the flow of interstitial fluid from the rete capillare capsulare at the inner side of the renal capsule, towards the district of progenitor cell recruitment located at the outer side of the nephrogenic zone. Unfortunately, data about the molecular composition of the here contained interstitial fluid, such as pH, respiratory gas, nutrients, or metabolic products, is not available.

When orientated histological sections of a fetal human kidney are viewed, we can observe a CD ampulla, which lines vertically through the nephrogenic zone up to the inner side of the renal capsule (Figs. 1 and 4). Earlier investigations revealed that the tip of the CD ampulla does not contact the inner side of the renal capsule but stays in an average distance of 32 µm [8]. In the space between, only 2 to 3 cell layers of mesenchymal progenitor cells are detected. Histochemical labeling in the rodent and fetal human kidney shows that the CD ampulla tip is fixed by microfibers and so is not randomly positioned [26, 27]. Labeling by Soybean Agglutinin (SBA) reveals that these fibers originate at the inner side of the renal capsule, cross the underlying progenitor cell layers vertically to end at their final position at the tip of a CD ampulla. The SBA label detected on the microfibers is unique, due to the fact that it does not colocalize with collagen type I, III, IV, V, or VI, laminin, fibronectin, or tenascin. The demonstrated microfibers obviously influence the attitude of renal progenitor cells. For example, collagen type V microfibers stimulate the migration of contacting cells, while collagen type V molecules support their stabilization during nephrogenesis [28].

It was further shown in the rodent kidney, that the cell bodies of nephrogenic progenitor cells do not touch but are separated by a striking interface from the basal aspect of epithelial progenitor cells integrated in the tip of a CD ampulla [27]. A special fixation including contrasting with cupromeronic blue, ruthenium red, or tannic acid for the electron microscopy made it possible to reveal previously not visible textured extracellular matrix. Remarkably, the projections of the nephrogenic mesenchymal progenitor cells cross the interface at this point, penetrate the plasma membrane at the basal aspect of epithelial progenitor cells to establish contacts via tunneling nanotubes. This special contact within the center of a nephrogenic niche enables site-specific cell to cell communication and the exchange of poorly soluble morphogenic molecules such as Bmps, Wnts, or Shhs.

### The CD ampulla at the medial limit

The spatial proximity between the medial aspect of the developing nephron and the CD ampulla is striking. Originally, a CD ampulla derives from the ureteric bud. However, in the fetal human kidney during late gestation, it is spatially separated from the start of organogenesis [29]. The current data shows that a CD ampulla is restricted to the nephrogenic zone. Its neck is seen at the distal end of a collecting duct tubule, while its dilated conus and head cross the nephrogenic zone (Figs. 1 and 4). Moreover, the tip is positioned near the inner side of the renal capsule. In accordance with the outer shape of a CD ampulla, the lumen is wide in the head and upper conus but decreases towards the neck. Surprisingly, data about the molecular composition of the contained clear fluid is not available. At a later stage, a CD ampulla is covered by a single-layered prismatic epithelium. The apical side faces the lumen, while the basal side rests on a basement membrane exhibiting a special reticular meshwork at the tip [30]. The size of a CD ampulla is remarkable. Previous research revealed that it has a length between 80 and 100 µm and has an average diameter of 68 µm [8]. Depending on the current status of nephron development, a CD ampulla first elongates towards the renal capsule and then branches at the tip. In this way, it exhibits a vertical but slightly slanted course.

When the formation of a nephron begins, the tip of the CD ampulla represents the epithelial part of the nephrogenic niche. During subsequent development, the medial aspect as well as the pretubular aggregate (Fig. 4a), renal vesicles (Fig. 4b–f), and comma- (Fig. 4g–i) and S-shaped bodies (Fig. 4j–l) first faces the head, then the conus and later the neck of the CD ampulla in a stage-specific fashion. Thus, not only the medial aspect of the respective nephron anlage but also the related sector of the CD ampulla changes with each developmental step.

The meeting of mesenchymal and epithelial cells within a nephrogenic niche, the aggregation of induced nephrogenic mesenchymal progenitor cells, and the subsequent formation of a pretubular aggregate first occur close to the tip and subsequently at the head of a CD ampulla (Figs. 1a and 4a). Regarding these initial stations, we can recognize that the cell bodies of the developing nephron do not touch the tip or the head of the CD ampulla. Instead, a clear interface separates both. However, at the section border between the head and the conus of the CD ampulla the clear interface is replaced by a close adhesion. This marks the mounting point between the future connecting tubule (CNT) and the forthcoming transverse separation between the pretubular aggregate and the primitive renal vesicle (Fig. 4b, c).

As previously mentioned, the formation of the mature (Fig. 4d), extending (Fig. 4e), and extended (Fig. 4f) renal vesicles proceeds in the underlying area of nephron shaping. The medial aspects of these stages faces the upper conus of the CD ampulla. During this period, the proximal pole of the renal vesicle is positioned close to the subjacent connecting tubule of a previously developed nephron. As a consequence, a spatial expansion of the renal vesicles towards the underlying maturation zone is blocked due to already established structures. However, it enables a radial extension of the area of nephron shaping including the above-positioned district of progenitor cell recruitment and the renal capsule. During this process, the tip and head of the CD ampulla maintain their shape, while the conus elongates in parallel to the vertical extension of the renal vesicles. The medial aspect of the renal vesicles keeps a close structural relation with the conus of the related CD ampulla. However, during the development of the comma-shaped body (Fig. 4g–i) and the S-shaped body (Fig. 4j–l) the earlier seen close adhesion between the medial aspect of the renal vesicles and the conus of the CD ampulla is transforming into a vertically lining interstitial cleft.

During the development of the comma-shaped body, the future connecting tubule is integrated into the epithelium at the border between the head and conus of the CD ampulla (Fig. 4g–i). At the same time, the tubule anlage of the comma-shaped body elongates by winding. This again causes the interstitial cleft between the medial aspect of the comma-shaped body and the conus of the CD ampulla to elongate towards the interior of the comma-shaped body. When the S-shaped body arises, the conus of the CD ampulla takes up a slightly concave shape. This enables the development of a congruent structural relation between the medial aspect of the early (Fig. 4j), mid (Fig. 4k), and late (Fig. 4l) S-shaped bodies and the conus of the CD ampulla. At the same time, the anlage of the glomerulus further develops at the proximal pole. However, the ongoing winding of the tubule anlage causes the glomerulus anlage to turn further towards the lower conus and neck of the CD ampulla.

### The structural barrier at the proximal pole

The S-shaped body, as the last stage of nephron anlage, is released at the lower limit from the nephrogenic compartment located in the nephrogenic zone to the subjacent maturation zone for the transformation into the lasting nephron. During this step, the typical morphological as well as physiological features of the glomerulus become visible. This includes the expansion and differentiation of the tubular portions and the descent of the intermediate tubule (Henle’s loop) [9].

The data presented here shows that the proximal pole of the S-shaped body rests near the subjacent connecting tubule (CNT) of an earlier developed nephron (Fig. 1k, l). Performed measures further demonstrated that the distance between the renal capsule and the proximal pole of the S-shaped body in gestational controls is 150 µm reflecting the vertical width of the nephrogenic zone [5, 13]. However, current data indicates that this parameter can be decreased, when the measure is performed, for example, at a smaller comma-shaped body or an even smaller extending renal vesicle. Therefore, the width of the nephrogenic zone is not a constant parameter, but rather variable, and equally important as, it depends on the presently available stages of nephron anlage. Consequently, the proposal is to measure the current width of the nephrogenic zone in the fetal human kidney during late gestation as the vertical distance from the inner side of the renal capsule to the transversely lining connecting tubule of an earlier developed nephron. In this case, not only the width of the nephrogenic zone but also the current stages of nephron anlage at this point are recorded.

### The perforating radiate artery at the lateral limit

**Fig. 5:**
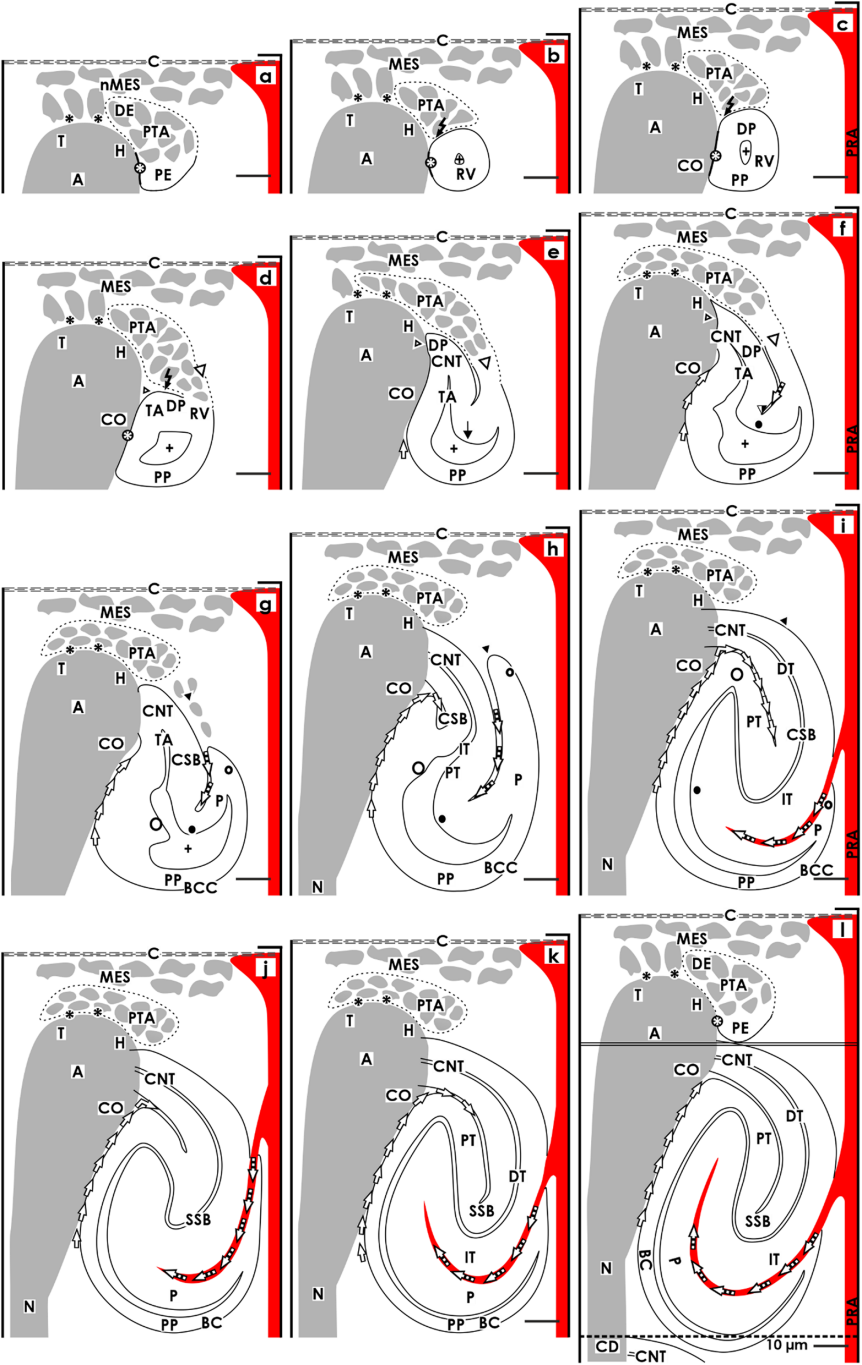
Graphical sketches show us the changing structural relations between the developing nephron and the perforating radiate artery in the fetal human kidney during late gestation. In the district of progenitor cell recruitment, the space between a perforating radiate artery (PRA, short black arrow) and the lateral aspects of **a** the pretubular aggregate (PTA), **b** the mesenchymal to epithelial transition, and **c** the primitive renal vesicle (RV) is filled with loose mesenchyme. However, in the underlying area of nephron shaping, this distance becomes smaller during development of the **d** mature, **e** extending, and **f** extended renal vesicle. When the **g** early and **h** mid-late comma-shaped body (CSB) are forming, an approximation of their lateral aspects with the perforating radiate artery is registered. Furthermore, a transverse interstitial cleft (black/white arrows) is opening at the deep lateral aspect of the **i** late comma-shaped body. In the **j** early, **k** mid, and **l** late S-shaped body (SSB), this cleft serves the invasion of an afferent arteriole via a short way towards the forming glomerular tuft. Renal capsule (C), collecting duct (CD) tubule, CD ampulla (A), and lumen of the arising nephron +

While the medial limit of the developing nephron shows close relations with the tip, head, conus, and neck of a CD ampulla (Fig. 4), the lateral limit faces loose mesenchyme and a neighboring perforating radiate artery (Fig. 5). This vessel originates from an interlobar artery and lines up to the border between the cortex and medulla to become an arcuate artery. From here, a cortical radiate artery elongates vertically through the inner, the mid, and, finally, the outer cortex. During this course, the afferent glomerular arterioles successively branch off, in order to contact the continuously younger generations of glomeruli. In the maturation zone, a cortical radiate artery transforms into a perforating radiate artery. This lines vertically through the nephrogenic zone to eventually anastomose with a vessel in the capsular capillary plexus of the covering renal capsule. However, a systematic investigation in the fetal human kidney during late gestation is not available.

Within the district of progenitor cell recruitment, one can see that a perforating radiate artery lines vertically within a certain distance along the lateral aspect of the pretubular aggregate, the mesenchymal to epithelial transition, and the primitive renal vesicle (Figs. 1a–c and 5a–c). It crosses the progenitor cell layers in a slight arc to then anastomose with the capsular capillary plexus. However, in the area of nephron shaping, the transverse distance between a perforating radiate artery and the extending renal vesicles (Figs. 1d–f and 5d–f), the comma-shaped body (Figs. 1g–i and 5g–i), or the S-shaped bodies (Figs. 1j–l and 5j–l) becomes smaller until a few micrometers are reached. This spatial decrease is mainly caused by the vertical and transverse expansion during the development of the comma- and S-shaped body. During this period, an elongation including a slight inclination at the CD ampulla is also observed, so that the increase of the area of nephron shaping is correlated with a 100 µm vertical elongation of the related perforating radiate artery.

During the development of the early comma-shaped body, the earlier mentioned two-layered mesenchymal progenitor cell strand, which maintained a connection with the overlying pretubular aggregate, is dissolving (Figs. 1g, h and 5g, h). As a consequence, an interstitial cleft is beginning to open at the lateral aspect of the early comma-shaped body (Fig. 1g, 5g). During the formation of the mid (Figs. 1h and 5h) to late (Figs. 1i and 5i) comma-shaped body, this cleft turns from a vertical to transverse position. Most remarkably, the opening of the interstitial cleft is turning during the development of the early (Figs. 1j and 5j), mid (Figs. 1k and 5k), and late (Figs. 1l and 5l) S-shaped body to its deep lateral aspect so that it is finally facing a neighboring perforating radiate artery. The same constellation was shown for the rodent kidney [22]. Although not experimentally proven, it is most probable that this close vicinity enables an afferent glomerular arteriole to invade the developing glomerular tuft via a short way in order for the capillaries and the mesangium to form.

The developing nephron as well as the surrounding structural limits within a nephrogenic compartment must be provided with nutrition and respiratory gas, while the harmful metabolites are removed. There are a number of issues in this case, such as the unavailability of data with the physiological microenvironment of the nephrogenic zone as well as the fact that the district of progenitor cell recruitment as the area of nephron shaping is under permanent morphological alteration and that the microvascular supply is under construction. It was recently demonstrated by immunohistochemical labeling, that the nephrogenic zone in the fetal human kidney is poorly vascularized, reflecting a bradytrophic microenvironment [31]. For the rodent kidney, it was shown that lacunae-like accesses exist at the inner side of the renal capsule. These are connected with a tunnel network, which is formed by projections of the smooth muscle cells in the tunica muscularis of the renal capsule [25]. It is yet to be explored, whether the here contained interstitial fluid serves to provide progenitor cells and the developing nephron with nutrition and respiratory gas.

### The interstitium at the developing nephron

It was shown that the formation of a nephron in the nephrogenic zone of the rodent kidney depends on coordinated morphogenic interactions between the ureteric bud-derived tips at the distal collecting duct tubules, nephrogenic progenitor cells, hemo-endothelial cells, and macrophages [12]. The interstitial/stromal progenitor cells expressing the Forkhead transcription factor FOXD1 or the Prorenin receptor play a mastering role in these processes [32]. However, only little is known about the related cellular and molecular interactions [33]. The transiently appearing extracellular matrix, which points to a special interstitial microenvironment at the developing nephron, must be commented on [34]. Unfortunately, comparable data for the fetal human kidney was not shown.

**Fig. 6:**
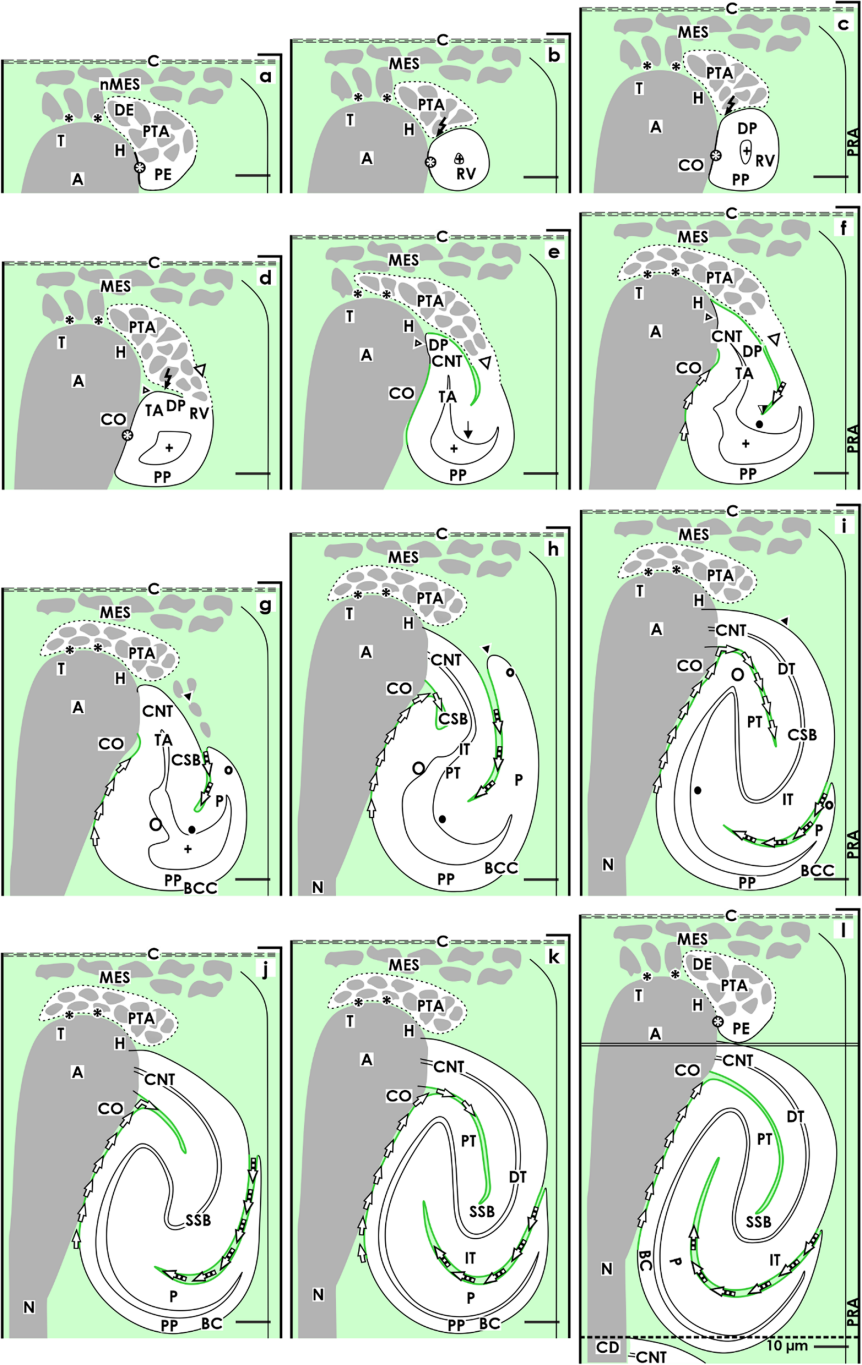
Graphical sketches depict the expanding interstitium, which envelops the developing nephron in the fetal human kidney during late gestation. In the district of progenitor cell recruitment, the interstitium is recognized at the inner side of the renal capsule, and between the mesenchymal progenitor cells. **a** In the nephrogenic niche, an interface is seen between the innermost layer of nephrogenic mesenchymal progenitor cells and the tip of the CD ampulla. This extends at the lateral aspect of the pretubular aggregate (PTA), **b** the mesenchymal to epithelial transition, and **c** the primitive renal vesicle (RV). In the area of nephron shaping, the interstitium is between the medial aspect of the **d** mature, **e** extending, and **f** extended renal vesicle. Furthermore, it covers the conus of the CD ampulla, where a vertical interstitial cleft forms. In parallel, between the progenitor cell strand (white arrow head) connecting the renal vesicles with the pretubular aggregate and the tubule anlage (TA) including the connecting tubule (CNT) a narrow interstitial cleft becomes visible. During the development of **g** the early comma-shaped body, the progenitor cell strand dissolves (black arrow head). As a result, during the formation of the **h** mid and **i** late comma-shaped body (CSB), the cleft opens near the lateral fold (black/white point). Due to the winding of the tubule anlage, the interstitial cleft turns from a vertical to transverse direction during the development of the **j** early, **k** mid, and **l** S-shaped body. Yet, it opens at the deep lateral aspect. In contrast, the interstitial cleft between the medial aspect of the S-shaped body lines first along the conus of the CD ampulla and then to the connecting and finally to the distal tubule segments before reaching the geometrical center. Renal capsule (C), collecting duct (CD) tubule, CD ampulla (A), perforating radiate artery (PRA, short black arrow), and lumen of the arising nephron +

The morphological data presented here reveals that the developing nephron in the fetal human kidney during late gestation is connected first at its distal pole via the connecting tubule (CNT) with the CD ampulla (Fig. 6e) and later via an afferent arteriole at the glomerulus arising at its proximal pole (Fig. 6l). Logically, the rest of the nephron surface is occupied by the earlier shown tissues (Figs. 3, 4, and 5). Depending on the current developmental progress, these establish contacts, form congruent surfaces or stay in a distinct distance. As a necessary consequence, the interjacent space is filled by the interstitium. The situation is further complicated by the fact that the interstitium is not homogenously composed but arises by the fusion of interstitia with different origins and ages. For example, underneath the renal capsule, in the district of progenitor cell recruitment, an interstitium of embryonic origin is present. This supports the spatial sorting, renewal of progenitor cells, targeted aggregation of induced nephrogenic progenitor cells, site-specific formation of the pretubular aggregate, mesenchymal to epithelial transition, and formation of the primitive renal vesicle (Fig. 6a–c). In contrast, in the underlying area of nephron shaping, the interstitium is newly synthesized to cover the continuously increasing surfaces of the renal vesicles (Fig. 6d–f) and comma- (Fig. 6g–l) and S-shaped bodies (Fig. 6j–l). The proximal pole of the respective stage of nephron anlage faces the interstitium at the connecting tubule of an earlier developed nephron, yet located in the underlying maturation zone.

An additional observation is that the contour as well at the proximal and distal poles as at the medial and lateral aspects of the developing nephron are changing in an asymmetrical manner. It is obvious that the same applies for the covering interstitium. To illustrate aspects of these complex relations, it is best to start at the nephrogenic niche. During the process of induction, the nephrogenic mesenchymal and the epithelial progenitor cells meet at the tip of a CD ampulla (Figs. 1a and 6a). Both partners exchange morphogenic molecules but are faced at this site by an interstitium exhibiting special microfibers and a striking interface [25, 30].

At the distal pole: During the aggregation of induced mesenchymal progenitor cells and the formation of the pretubular aggregate, it can be observed that the interstitial interface elongates from the nephrogenic niche at the tip towards the head of the CD ampulla, by facing the medial part of the pretubular aggregate (Figs. 1b, c and 6b, c). Later, it elongates as a cleft between the future connecting tubule of the extending renal vesicles and the progenitor cell strand connected with the proximal end of the pretubular aggregate (Figs. 1d, e and 6d, e). In the extended renal vesicle, the distal end of this cleft is detected due to elongation between the legs of the inner fold (Figs. 1f and 6f).

At the medial aspect: The distal pole of the primitive renal vesicle is mounted at the section border between the head and the conus of the CD ampulla (Figs. 1a–c and 6a–c). At this particular site, the clear interface develops in a close adhesion reflecting the link with the future connecting tubule. Underneath the contact site, a vertically lining interstitial cleft elongates between the medial aspect of the extending renal vesicles and the conus of the CD ampulla (Figs. 1d–f and 6d–f). During the development of the comma-shaped body, the process of internal folding causes that the interstitial cleft to change direction and expand along the connecting but also distal tubule portions (Figs. 1g–i and 6g–i). When the S-shaped body is forming, the cleft further extends along the intermediate and proximal tubule portions (Figs. 1j–l and 6j–l).

At the lateral aspect: A two-layered progenitor cell strand connects the primitive renal vesicles (Figs. 1c and 6c) up to the extended renal vesicle (Figs. 1f and 6f) with the above situated pretubular aggregate. However, during the transformation into the early comma-shaped body, this cell strand is dissolved (Figs. 1g and 6g). As a consequence, the enclosed interstitial cleft is opened. As a result, a fusion takes place with the loose mesenchyme surrounding the lateral aspect of the developing nephron and the neighboring perforating radiate artery. This again also causes the distal pole, the lateral aspect, the proximal pole near the transversely lining connecting tubule of an earlier developed nephron as well as the medial aspect of the currently developing comma-shaped body to be covered by a hoof-shaped bracket of interstitium (Figs. 1j–l and 6j–l).

At the proximal pole: During the formation of the comma-shaped body, the interstitial cleft at its deep lateral aspect changes direction from vertical to transverse, so that it eventually opens near the proximal pole (Figs. 1j–k and 6j–k). Yet, the opening of the cleft is positioned near the perforating radiate artery enabling the targeted ingrowth of interstitial cells including an afferent glomerular arteriole as the formation of capillary loops and the mesangium. Finally, when the late S-shaped body forms, the transverse interstitial cleft near the proximal pole shows a vertical elongation towards the distal pole enabling a crescent-like shape to arise (Figs. 1l and 6l). This again causes the proximal pole to be positioned close to the lower conus and neck of the CD ampulla.

## Discussion

**Fig. 7:**
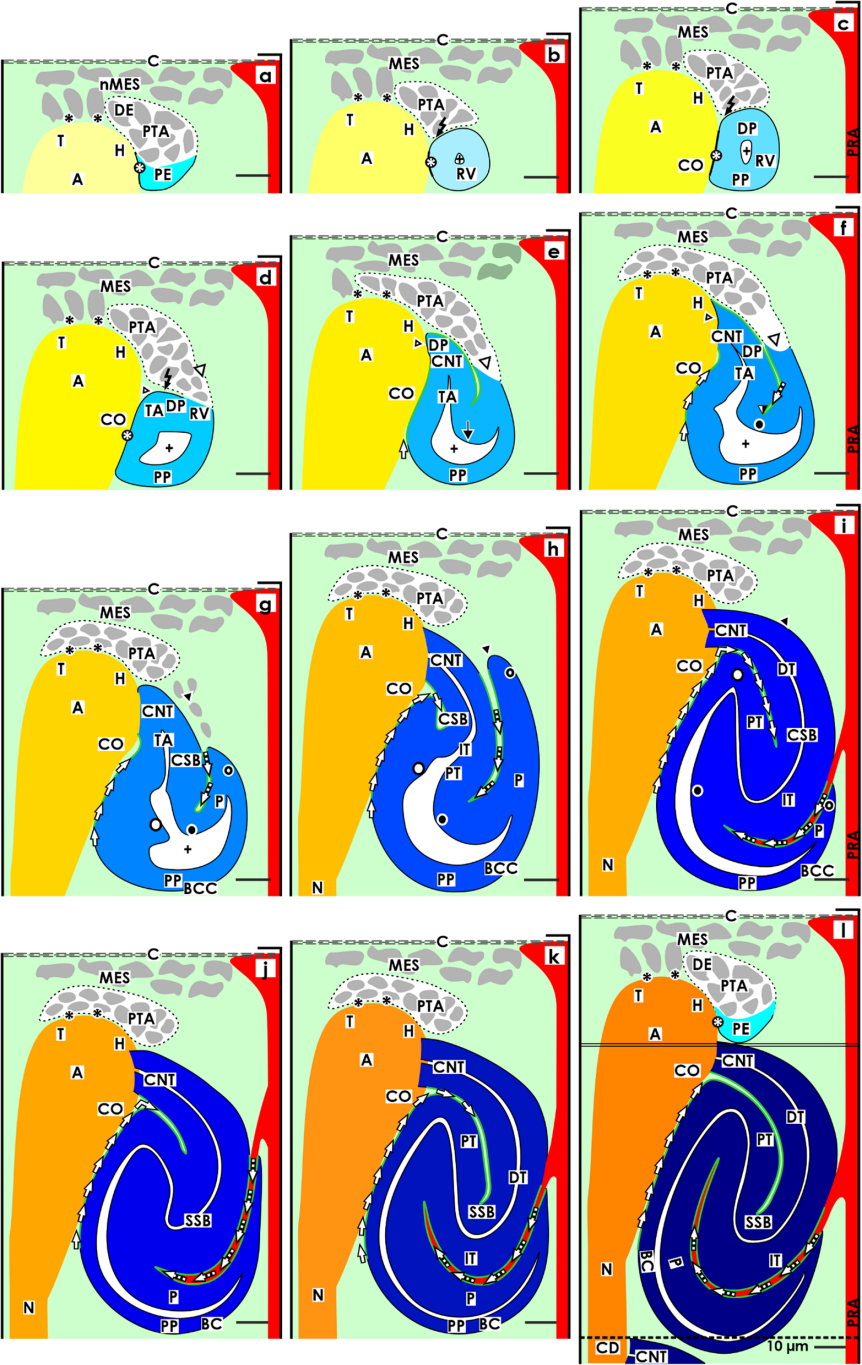
Merging of the graphical sketches 3 to 6 demonstrates the mutual changes between the developing nephron and its covering tissues in the fetal human kidney during late gestation. Depicted are the **a** nephrogenic niche and pretubular aggregate (PTA), **b** the mesenchymal to epithelial transition, and **c** primitive renal vesicle (RV). In addition, the **d** mature, **e** extending, **f** extended renal vesicle, the **g** early, **h** mid, and **i** late comma-shaped body (CSB) and the **j** early, **k** mid, and **l** late S-shaped body (SSB) are shown. The sequence of sketches illustrates that not only the stages of nephron anlage, but also the covering tissues and the interjacent interstitium are changing. Renal capsule (C), collecting duct (CD) tubule, CD ampulla (A), perforating radiate artery (PRA, short black arrow), and lumen of the arising nephron +

The pioneering morphological research into nephron formation of the human kidney in early organogenesis was first performed 100 years ago [34]. However, information about the special location [8, 9] and the shaping [10] of the developing nephron in the fetal human kidney during late gestation has only been discovered over the last few years. As a subsequent step, the investigation presented here reveals the mutual patterning between the stages of nephron anlage (Figs. 1 and 3) and the covering tissues. These are the renal capsule at the top, the elongating CD ampulla at the medial aspect (Fig. 4), the perforating radiate artery at the lateral aspect (Fig. 5), and finally, the interjacent interstitium (Fig. 6). To summarize the different backdrops, the data generated in Figs. 2, 3, 4, 5, and 6 have been merged to form Fig. 7.

### Extended workbench for a developing nephron

The generated results reveal that the workbench for a developing nephron is changing. It starts in the district of progenitor cell recruitment, which is represented by the nephrogenic niche, mesenchymal to epithelial transition, pretubular aggregate, and the primitive renal vesicle. Covering tissues can be recognized in the renal capsule, the head of the CD ampulla, and a short segment of the radially lining perforating radiate artery. Regarding the generated illustrations (Figs. 2a–c and 7a–c), only moderate structural alterations can be recognized during the start of nephron formation. In contrast, within the area of nephron shaping, a comparably significant structural accession is registered. This concerns the mature, extending and extended renal vesicles (Figs. 2d–f and 7d–f) and continues with the comma- (Figs. 2g–i and 7g–i) and S-shaped bodies (Figs. 2j–l and 7j–l). As covering tissues are identified the elongating conus and neck of the CD ampulla (Fig. 4), the sprouting perforating radiate artery (Fig. 5) and the interjacent interstitium (Fig. 6). This structuring opens up the possibility to compare the kidneys of preterm and low birth weight babies with gestational controls on the basis of objective criteria and by simple morphometric methods. It enables us to, for example, identify whether the ratio between the district of progenitor cell recruitment and the area of nephron shaping is maintained or whether it is untypically changing in the nephrogenic compartment, as well as to confirm which of the contained structural compounds have developed normally or abnormally, respectively. The almost true to scale sketches provided here can be used as a template to document the precise site and respective alteration.

### Nephron formation at the outer edge

Most available molecular biological data focus on nephron development during early organogenesis [16, 17, 35]. However, the situation of a developing nephron in the fetal human kidney during late gestation is different.

First of all, it takes place in the structured outer cortex of a lobe, is restricted to the nephrogenic zone, and proceeds perpendicular to the renal capsule [9].

Second, with each generation of newly formed stages of nephron anlage, the cessation of nephrogenesis as a natural process at birth comes closer [36].

Third, the formation of a nephron takes place spatially organized in a nephrogenic compartment [10].

Fourth, it is unknown what happens to the remaining pretubular aggregate, when the primitive renal vesicle is separating (Figs. 1, 2, 3, 4, 5, and 6b–g).

Fifth, the microscopic images presented here (Fig. 1) and the related series of sketches show that the starting nephron is not the sole structure, which develops in a nephrogenic compartment (Figs. 3, 4, 5, 6, and 7).

The top of a developing nephron is covered by the renal capsule and a set of progenitor cells [15, 16, 34]. At its medial aspect, the nephron is surrounded by the elongating ureteric bud-derived CD ampulla (Fig. 4), while at its lateral aspect, it is surrounded by a sprouting perforating radiate artery (Fig. 5). In the interjacent space, the interstitium is expanding (Fig. 6). The produced sequence of illustrations depicts that this is not a static frame. Instead, in concert with the successively developing stages of nephron anlage, the covering tissues are subjects of a permanent structural change (Fig. 7). It appears that the micromovements of the covering tissues, shown here, direct the precise positioning, correct orientation of the proximal–distal axis, and tailored shaping at the medial and lateral aspects of the developing nephron. This in turn enables to link the arising nephron in the right place and at the right time with the CD ampulla via the future connecting tubule (Fig. 7e). Furthermore, by the precise positioning, the connection between the arising glomerulus and the perforating radiate artery is prepared so that an afferent glomerular arteriole can invade via a short way (Fig. 7i).

Regarding the complex mutual patterning, it is important to ask ourselves, whether not only the nephron but also its covering tissues are potential targets of noxae impairing nephrogenesis. The fact is that, as well as the renal capsule [37], the epithelial progenitor cells in the CD ampulla [38], the formation of microvessels [39] as the arising interstitium [33, 40] are controlling instances for the formation of a nephron.

### Numerous unanswered questions

The microanatomical description of the mutual patterning between the developing nephron and its covering tissues supports the screening of microscopic specimens, the precise allocation of marker molecules, or the assessment in molecular pathology and renal pediatric research. Of particular analytical importance is the developmental point at which the renal vesicle is translocated from the district of progenitor cell recruitment (Fig. 7a–c) to the area of nephron shaping (Fig. 7d–l). This step results in little explored configurations between the developing nephron and its covering tissues to arise at the proximal and distal poles as well as at the medial and lateral aspects.

Surprisingly close structural relations can be noticed between the CD ampulla and the medial aspect of the developing nephron, for example. This starts at the tip of the CD ampulla during the process of induction in the nephrogenic niche and the formation of the pretubular aggregate (Fig. 7a), and continues during the mesenchymal to epithelial transition (Fig. 7b) and the development of the primitive renal vesicle (Fig. 7c), which is forming along its head. After this, at the border between the head and conus of the CD ampulla a remarkable adhesion becomes visible, instead of an interface. This serves as the first adhesion and then the link develops with the mature, extending, and extended renal vesicles via the future connecting tubule (Fig. 7d–f). During the further development, the medial aspect of the comma-shaped body (Fig. 7g–i) shifts near the elongating conus of the CD ampulla. In parallel, the adhesion is replaced by an interstitial cleft. Finally, during the formation of the S-shaped body (Fig. 7j–l), its proximal pole including the arising glomerulus is positioned near the conus and neck of the CD ampulla. In contrast, the lateral aspect of the developing nephron is built up in a quite different way facing the extending interstitium so that an afferent glomerular arteriole originating from the perforating radiate artery can meet the proximal pole via a short way. Hence, the challenge is to investigate the morphogenic proteins, which create the asymmetrical shape of the nephron at the medial and lateral aspects [41]. A further task is to investigate the expression of stage-specific proteins [35, 42]. To gain knowledge on the transiently, constitutively, and facultatively expressed molecular markers will support the investigation not only into normal development but also atypical expression and pathologic alterations evoked by the impairment of nephrogenesis [43].

### Concrete proposal for starting with analytical work

The results presented here suggest the value of further investigating the earlier described traces left by the impairment of nephrogenesis. Of particular interest is to identify, which tissue or tissues cause the reported decrease in width of the nephrogenic zone in the kidneys of preterm and low birth weight babies [5]. The same applies to the described lack of basophilic S-shaped bodies located here [6]. Thus, the proposal is to elaborate on our knowledge of whether it reflects an abrupt, diffuse, or rather focused damage. In addition, we can identify, whether other than the S-shaped bodies and whether pathologic alterations on the here illustrated covering tissues are observed. It would also help to determine the intactness of structures. In this context, we must focus on the described cell and molecular biological processes such as adhesion, distancing, winding, cleft formation, or exchange of morphogenic proteins between the stages of nephron anlage and its covering tissues. Only the consequent analysis of gestational controls and affected kidneys of preterm and low birth weight babies will enable us to obtain more insights into the pathogenic mechanisms involved and will open up new possibilities to think about strategies, which create a reliable therapeutic prolongation of nephrogenesis [44].

## Conclusions

The present microanatomical investigation deals with the potential cell targets of noxae impairing nephrogenesis in preterm and low birth weight babies. For the first time, the focus is directed not only on the developing nephron in the fetal human kidney during late gestation but also on its covering tissues. The almost true to scale sketches, which have been produced and a series of practical hints guide the screening of microscopic specimens, help to precisely allocate marker molecules and indicate developmental key points, which might be of special importance for the molecular pathological assessment. In sum, the contribution supports the search for molecular traces left by the impairment of nephrogenesis in preterm and low birth weight babies on the stages of nephron anlage and their covering tissues.

## Data Availability

The data sets generated and/or analyzed during the current study are not publicly available due to ongoing research but are available from the corresponding author on reasonable request.
